# Determinants of the early initiation of breastfeeding in the Kingdom of Saudi Arabia

**DOI:** 10.1186/s13006-019-0207-z

**Published:** 2019-04-02

**Authors:** Adam E. Ahmed, Osama A. Salih

**Affiliations:** 1 College of Agriculture and Food Sciences, King Faisal University, Al-Hafouf, Kingdom of Saudi Arabia; 2 Department of Agribusiness and Consumer Sciences, University of Khartoum, Shambat, Sudan; 3 Department of Community Health, College of Applied Medical Sciences, King Saud University, Riyadh, Kingdom of Saudi Arabia

**Keywords:** Breastfeeding, Determinants, Early initiation, Saudi Arabia

## Abstract

**Background:**

National surveys in the Kingdom of Saudi Arabia continue to show rates of breastfeeding below global recommendations. Delay of timely or early initiation of breastfeeding is common in the community. Current approaches are not addressing the major constraints. Objectives of this study were to study the pattern and determinants associated with the early and delayed initiation of breastfeeding practices among infants aged less than 24 months. Also to assess regional differences to facilitate targeted actions.

**Methods:**

This cross-sectional study was conducted from May to August 2016, based on a sample of 1700 mothers of children aged less than 24 months. The sample was randomly selected from over 165 health centers distributed across the country, at least 33 centers in each of the five geographical regions. World Health Organization standardized questionnaire for infant and young child feeding indicators was used to prepare structured questionnaire (in English translated later to Arabic) to collect the information on breastfeeding along with general sociodemographic data.

**Results:**

The breastfeeding initiation rate was 97.3% (1559/1700 mothers). Only 8.3% (141) of mothers never breastfeed their children. Early initiation of breastfeeding within 1 h of birth was 43.6% (742) among all mothers surveyed while, breastfeeding initiation rates for 1-24 h and that for more than 24 h were 27 and 21% respectively. The overall prevalence of early initiation of breastfeeding (43.6%) is considered ‘fair’. Regional variation showed poor prevalence 26% (79/300 mothers) in Northern region; fair 38.4% (192/500 mothers), 45% (135/300 mothers), 49% (148/300 mothers) in the Central, Western and Eastern regions respectively; and good 63% (188/300 mothers) in the Southern region. Significant associations were indicated between early initiation of breastfeeding and mode of delivery, knowledge of the mother about the right time for early initiation, receipt of breastfeeding information, the region of residence, and educational level of the mother.

**Conclusion:**

Whilst some barriers to breastfeeding initiation manifest similarity across the regions some factors were context-specific thus, tailored interventions are imperative. Appropriate behavior change interventions, are needed attain optimal breastfeeding practices.

## Background

The Kingdom of Saudi Arabia (KSA) has a population of 32 million and a male:female distribution of 57:42% respectively [1, 2]. At least 24.8% of the total population are age less than 15 years while the people who ranges between 15 and 64 years old represent 72%, and only 3.2% are older than 65 years. About one quarter (24.8%) of the population live in Riyadh, the capital city of KSA, the Western region (Jeddah, Makkah and Taif) is inhabited by another 24%; the Eastern province by 13.3% and the remaining spread over the rest of the country [2].

What had been customarily a breastfeeding culture in Saudi Arabia, was disrupted with the great influx of oil wealth in the 1970’s and 1980’s [3]. The country witnessed massive advancements in socioeconomic status and became a target for breast milk substitutes industry. This coupled with other social and economic factors has caused considerable change in the original pattern of breastfeeding in recent decades in the country [3].

Breastfeeding is recognized globally as a vital public health issue with vast health, social and economic implications. It is manifested that, the recommendation for optimal breastfeeding practices in the Kingdom of Saudi Arabia (KSA) is based firstly on the Holy Quran, and then the commitments to the objectives of the global strategy for the Infant and Young Child Feeding (IYCF) and the recommendations of the World Health Organization (WHO) and United Nations Children’s Funds (UNICEF) [3–5]. Yet, current surveys remain to show that, poor breastfeeding practices (e.g. the delay of the early initiation of breastfeeding) are common in the Saudi communities [6–8].

The benefits of initiation of breastfeeding within 1 h of birth has long been recognized, understood and well documented [9–11]. The WHO and the UNICEF recommend that children initiate breastfeeding within the first hour of birth and be exclusively breastfed for the first 6 months of life [5]. Recent global reports [5, 12] indicate that, most of the world’s newborns are waiting too long to begin breastfeeding, and in 2017 alone, an estimated 78 million newborns had to wait more than 1 h to be put to the breast.

Recent meta-analysis [13, 14] investigate the links between the delayed breastfeeding initiation and infant survival, results indicated that newborns who began breastfeeding between 2 and 23 h after birth had a 33% greater risk of dying compared with those who began breastfeeding within 1 h of birth [13]. Also, among newborns who started breastfeeding 24 h or more after birth, the risk was more than twice as high.

When breastfeeding is delayed after birth, the consequences can be life-threatening, and the longer newborns are left waiting, the greater the risk [5]. Early initiation of breastfeeding (EIBF) is an indicator defined according to WHO as the proportion of children born in the last 24 months who were put to the breast within 1 h of birth [10, 11]. However, for initiating breastfeeding within the first hour of life, mothers need adequate support, direction and encouragement on positioning and feeding their newborns. Nevertheless, it is indicated that once the decision to breastfeed is made, different social and behavioral factors influence the duration of breastfeeding and its exclusivity [15]. For instance, in some countries there are cultural practices that involve feeding newborns supplemental foods or drinks, or having an elder family member give the newborn a specific food or liquid, or having health professionals routinely give the newborn a specific liquid, such as sugar water or infant formula [16].

Other factors and reasons why newborns are missing out on breastfeeding in the first hour of life include outdated practices in health facilities such as immediate separation of mothers and babies after birth. The dearth of knowledge about breastfeeding after a caesarean delivery that can lessen the probability of immediate skin-to-skin contact and hence reduce the early initiation of breastfeeding [17, 18].

In the context of the Kingdom of Saudi Arabia, a national infant and young child feeding/breastfeeding policy has been officially adopted/approved by the government [3]. Yet, there is presently an absence of a comprehensive surveillance system that shares and compiles data nationally, lack of community-based support centers easily accessible to mothers and absence of strategic plans for national implementation of IYCF. Moreover, the national Saudi Code of Marketing of Breast Milk Substitutes needs updating and revision [3].

The current practice of breastfeeding Saudi infants showed high (> 90%) prevalence of breastfeeding initiation at birth [19–23] compared to about 31% in the 1990s [24]. However, the initiation of breastfeeding rate tends to decline dramatically to low rates, because of the influence of many factors including (among many) mother’s age and education, parity and rooming in. The majority of the available literature regarding breastfeeding in KSA focuses on the rate of initiation rather than timely or early initiation of breastfeeding. For instance, a nationwide nutritional survey (2004–2005) of a sample of 5339 Saudi children was performed as part of the Health Profile for Saudi Children and Adolescents Projects. Results showed that, 4889 (91.6%) received breast milk at birth however, initiation of breastfeeding was delayed beyond 6 h after birth in 28.1% of the infants [25]. A cross-sectional observational study in Saudi Arabia in 2015 revealed that, 94.4% of the Saudi mothers were successful in initiating breastfeeding on the first day of delivery [19]. According to El Mouzan et al. and Al Juaid et al. [25, 26] initiation rates among Saudi mothers were high (mostly above 90%), but a few studies reported low rates of timely initiation (within the first hour).

Regarding the prevalence of the early or timely initiation of breastfeeding, the official figure reported by the World Breastfeeding Trend Initiative (WBTi) was 11.7% [3, 27]. A recent study in Mecca, Saudi Arabia showed that, early initiation of breastfeeding (< 1 h) was observed in 38.1% mothers, and that 42.7% mothers delayed the initiation of breastfeeding for > 1 h [28].

It is clear from the modest literature available regarding breastfeeding in KSA that, there is inconsistency in the outcomes of the studies tackling the issue of breastfeeding practices in KSA but particularly, the early initiation of breastfeeding. None of the published literature were able to diagnose and explain the genuine reasons behind poor timely/early initiation of breastfeeding in the kingdom of Saudi Arabia. There were many literature gaps that need critical investigation regarding the transition shift in behavioral and social norms of the people as well as the not conforming to the international health and nutrition agreements.

## Methods

After obtaining the required study approvals, the research team conducted this comprehensive cross-sectional, in-depth, health facility-based survey of Saudi mothers with infants less than 24 months of age from May to December 2016. The study was performed at primary health care centers (PHCCs) in five major cities (Riyadh, Hail, Jazan, Jeddah and Dammam) representing the different geographical regions (Central, Northern, Southern, Western and Eastern) of the Kingdom of Saudi Arabia (KSA).

Sample of 1700 mothers of infants aged 0 to less than 24 months were selected from the designated cities. A network of over 500 PHCCs is widely distributed all over the country, each PHCC has a well-defined catchment area and population therefore, each PHCC was considered as a cluster. Using a stratified multistage cluster sampling design [29], thirty-three (33) primary health care centers were selected from each region both from urban and rural areas (26 urban and 7 rural centers) based on the urban/rural ratio of 80/20 reported by the Saudi general authority for statistics [2]. More specifically, at least one PHCC located in each of the north, south, east, west and central areas of each region was randomly selected after stratification by population density, socioeconomic status and geographical location was carried out. Mothers fulfilling the eligibility criteria were enrolled in the study from each health center during the data collection period until the required sample size was reached.

The inclusion criteria were; mothers aged 15–49 years, who were attending one of the designated PHCCs, having a child less than 24 months of age, and being Saudi national. There were no distinctions made for the inclusion criteria regarding the body mass index of the mother and/or whether the child was term or preterm.

While exclusion criteria were; mothers with child older than 24 months and mother who had twins. The eligible mothers were approached in person and invited to an individual interview after they had received a proper orientation. Participants were fully informed about their rights to participate, and the right to withdraw, without any consequence, at any time prior to or at any point, during or after the start of the interview. Written informed consents were obtained from all participants and confidentiality was assured. Participants were assured that any information provided will be used only for research purposes.

### Data collection

The in-depth interviews with the selected mothers in each PHCC was carried out by experienced Arabic-speaking (Saudi national), female, health personnel (nurses and dietitians) and medical staffs (doctors,) working at each PHCC. Average of twenty to thirty data collectors were selected from the working staff in each region with the help and permission of the local health authorities. Specially designed training programs (2 days training) on the study tools were conducted by the investigators for the data collectors in each region prior to field survey. The data collection was carried out using an interview-administered questionnaire. The questionnaire was prepared in English with guidance from the WHO [4] standardized questionnaire for infant and young child feeding indicators. The questionnaire was translated into Arabic with back translation to check for reliability.

A Pilot survey was carried out among thirty-two Saudi mothers in four randomly selected health centers in Riyadh region, representing the four geographical locations of the city, with the purpose of testing the questionnaire which had been designed, and the pilot study participants were excluded from the final study sample. The generated data from the pilot survey was processed and analyzed, accordingly, questionnaire was revised, modified and edited. The questionnaire was checked for item flow, order, skip patterns, timing, and time needed for completion. The estimated time for completion was approximately 20 min. The questionnaire had three components: demographic data (for the infants, mothers and fathers) including age, education and employment; breastfeeding practice related data, including initiation of breastfeeding, exclusive breastfeeding, reasons and barriers for not or delayed initiation of breastfeeding, prelacteal (defined as any food except mother’s milk provided to a newborn infant before initiating breastfeeding), type of milk given, introduction of solid or semi solid food, and on demand feeding; and health service related data, including mode of delivery, pre and postnatal services, feeding discussion during antenatal checkup, receiving breast milk substitute advertisements and others.

### The structure of the questionnaire

This in-depth questionnaire used for collecting the study data consisted of the following main units, the first unit (comprised of two sections). Section A, for collecting socioeconomic characteristics [age (month and year), profession, level of education, marital status and monthly income] for the household head, mother and or caregiver. Section B, for the child background information [name, gender, date of birth, age, sex, method of delivery, birthweight (health card) and age of the previous child if any]. Additional questions were also included within this unit such as family size, whether the mother is working fulltime, part-time or not working and how many hours the mother stays away from the child (if she is working). In addition, questions regarding the child’s health situation (presence or absence of chronic diseases), and the status of vaccination situation were also enquired. The second unit of the questionnaire collected information regarding the early initiation of breastfeeding (EIBF). The following questions were asked to mothers: did you ever breastfeed (child name)? If the answer was “Yes”, the following questions were asked, how long after birth did you first put (child name) to the breast? The answers were reported as follows, immediately after birth; less than 1 h; less than 24 h (number of completed hours from 01 to 23 was recorded); 24 h or more (number of completed days was record). If the answer was “No” for the question ‘did you ever breastfeed the child?’, mothers were asked about the reason for not breastfeeding the child, then responses were recorded (baby sickness, mother sickness, have not enough milk, breastfeeding is too painful, breastfeeding is too stressful, body image, returning to work soon and other reasons). If the mother delayed breastfeeding for “less than 24 hours” or “more than 24 hours” she was then asked for the reason(s), and responses were then recorded (child given prelacteal feed, type of delivery, place of delivery, late discharge from the nursery, mother sickness, cultural factors and/or other reasons). The third unit of the questionnaire concerned with the administration of the infant and young child feeding. Questions in this unit were “what was your baby’s first feed?” (breast milk or colostrum, infant formula, herbs water, Glucose water, dates water, plain water and others). Mothers were also asked whether the child was breastfed yesterday (day or night) and if the child was breastfed from his mother or other method was used to fed breast milk (breastfed by another mother, given breast milk by spoon, cup, bottle or some other way). Mothers were also asked if the child was given oral rehydration solution (ORS) the day before the interview during a day and night? The other two units of the questionnaire were the knowledge attitudes and practices unit, and prenatal and postnatal care unit.

### Data management

For technical and logistic reasons, the field surveys were conducted in the five regions of the country, one at a time, during the period from May to August 2016. In the process of the preparation for the study surveys, the study team held orientation meetings with the health officials at the Federal Ministry of Health (FMOH), followed by a series of similar meetings with officials and the staff of the “women and child health” departments at the regional Ministries of Health during the pre-visits conducted to each region. These meetings reflected to the regional Ministries, the commitment and approval of the study by the FMOH, demanding facilitation and coordination with the study team. Moreover, updated information about the population density, socioeconomic status, geographical locations, characteristics of the local population, and logistic issues were collected, shared, discussed and managed through local committees specially formulated for the purpose of this study in each region. These committees assisted in selecting the data collectors and facilitating their training. At the end of the survey with mothers in each region, the investigators meet with the data collectors for the feedback on the survey process and to collect their finished work.

### Data analysis

The data generated by the questionnaire were assigned appropriate codes, processed, analyzed, interpreted and reported for each region and for the country. Analysis was performed on the collected data using the Statistical Package for the Social Sciences (SPSS) program, version 21.0.

The early initiation of breastfeeding indicator was calculated according to the following WHO equation [4];

$$ \mathrm{EIBF}=\left(\mathrm{children}\ \mathrm{born}\ \mathrm{in}\ \mathrm{the}\ \mathrm{last}\ 24\ \mathrm{month}\ \mathrm{who}\ \mathrm{were}\ \mathrm{put}\ \mathrm{to}\ \mathrm{breast}\ \mathrm{within}\ \mathrm{one}\ \mathrm{hour}\ \mathrm{of}\ \mathrm{birth}/\mathrm{children}\ \mathrm{born}\ \mathrm{in}\ \mathrm{the}\ \mathrm{last}\ 24\ \mathrm{month}\mathrm{s}\right)\ \mathrm{X}\ 100 $$

The delayed initiation rate of breastfeeding was calculated using the following equation;

$$ Delayed\ EIBF\ \left(\%\right)=100-\%\mathrm{EIBF} $$

### Statistical analysis

Statistical analyses were done with SPSS software (Statistical Package for Social Sciences; IMB Corp., Armonk, NY, USA), version 21. The chi-square (*X*^2^) test was used to find the association between categorical groups as a preliminary test for regression analysis. Multiple logistic regression was performed to find the possible determinants of delayed early initiation of breastfeeding by the adjusted odds ratio (AOR) and 95% confidence interval (CI) for different independent variables. To determine the possible factors associated with delaying the early initiation of breastfeeding, two groups were compared: mothers who initiated breastfeeding early (< 1 h; *n* = #) and mothers who delayed breastfeeding (> 1 h; *n* = #) in the logistic regression. A *p* - value of < 0.05 was considered statistically significant.

Chi-square test (*X*^2^) was used to test the degree of association between the early initiations of breastfeeding (timely vs delayed initiation) against each of selected predictors. These predictors include: methods of delivery (vaginal vs cesarean), location (urban vs rural); receiving of antenatal care and postnatal care, breastfeeding information (yes, no); child order (first vs second and above), mother knowledge about the right time for initiating breastfeeding (yes, no), educational level (secondary and below vs graduates and postgraduates); mother age (< 25 year vs ≥ 25 years); occupation (working and students vs housewife), the region of residence (Central, Eastern, Western, Northern and Southern), infant birthweight at delivery (< 2.5 kg vs ≥ 2.5 kg).

Multiple logistic regression was used to identify the key determinants of early initiation of breastfeeding in the surveyed Saudi mothers. Predictors with chi-square (*p* - value of less than 0.05) were selected to be included in the multiple logistic regression and then after, running the regression analysis, only the significant predictors were included in the final multiple logistic regression analysis. The dependent variable in the multiple logistic regression was initiation of breastfeeding, which is binominal variable (timely vs delayed). Where “One” was assigned for mothers who initiate breastfeeding within 1 h of birth and “Zero” otherwise. Multiple logistic regression independent variables include infant method of delivery; mother knowledge about the right time for initiating breastfeeding; education level of the mother; receiving information about breastfeeding and region of residence.

## Results

### Demographic characteristics of the study population

As shown in Table 1 almost half of the mothers (48%) age was between 20 and 35 years and the other half (50.6%) were aged 30 years and above. While, 55.8% of fathers their age was between 30 and 40 years and 19.9% between 40 to 50 years. Results indicated that the majority of mother (66.5%) were housewives (unemployed). As for the fathers, the majority of them were working either as government employees (45.8%) or working in private sector (36.1%). It was clear from the collected data that, 49.5% of mothers and 41.4% of fathers had university level education. The marital status of the parents showed some differences particularly between divorced mothers (32.1%) and divorced fathers (18.6%).

**Table 1:** Demographic characteristics of the study population

Demographic characteristics	Region										KSA *n = 1700*	
	Central (Riyadh) *n = 500*		Southern (Jazan) *n = 300*		Eastern (Dammam) *n = 300*		Northern (Hail) *n = 300*		Western (Jeddah) *n = 300*			
	*n*	*%*	*n*	*%*	*n*	*%*	*n*	*%*	*n*	*%*	*n*	*%*
Maternal age												
15 – < 20 years	7	1.4	9	3.0	4	1.3	2	0.7	1	0.3	23	1.4%
20 – < 25 years	86	17.2	44	14.7	51	17.0	28	9.3	38	12.7	247	14.5%
25 – < 30 years	189	37.8	88	29.3	95	31.7	107	35.7	91	30.3	570	33.5%
30 – < 35 years	103	20.6	71	23.7	73	24.3	85	28.3	80	26.7	412	24.2%
≥ 35 years	115	77.0	88	29.3	77	25.7	78	26.0	90	30.0	448	26.4%
Mother work												
Housewife	368	73.6	170	56.7	210	70.0	200	66.7	182	60.7	1130	66.5%
Working Mothers	108	21.6%	106	35.3%	83	27.7%	88	29.3%	105	35%	490	28.8%
Student	24	4.8	24	8.0	7	2.3	12	4.0	13	4.3	80	4.7%
Mother education												
Illiterate	22	4.4	12	4.0	15	5.0	7	2.3	10	3.3	66	3.9%
Primary level	69	13.8	27	9.0	12	4.0	15	5.0	19	6.3	142	8.4%
Intermediate level	77	15.4	34	11.3	29	9.7	17	5.7	31	10.3	188	11.1%
Secondary level	119	23.8	69	23.0	106	35.3	91	30.3	76	25.3	461	27.1%
University level	211	42.2	158	52.7	138	46.0	170	56.7	164	54.7	841	49.5%
Postgrad level	2	0.4	0	0.0	0	0.0	0	0.0	0	0.0	2	0.1%
Mother marital status												
Married	450	90.0	293	97.7	294	98.0	295	98.3	287	95.7	1619	61.6
Divorced	48	9.6	6	2.0	6	2.0	5	1.7	13	4.3	78	32.1
Widowed	2	0.4	1	0.3	0	0.0	0	0.0	0	0.0	3	0.2
Father age												
20 - < 30 years	108	21.6	60	20.0	67	22.3	46	15.3	49	16.3	330	19.4
30 - < 40 years	273	54.6	159	53.0	159	53.0	185	61.7	172	57.3	948	55.8
40 - < 50 years	94	18.8	62	20.7	62	20.7	57	19.0	63	21.0	338	19.9
50 - < 60 years	23	4.6	15	5.0	7	2.3	10	3.3	16	5.3	71	4.2
> 60 years	2	0.4	4	1.3	5	1.7	2	0.7	0	0.0	13	0.8
Father work												
Government employee	244	58.8	141	47	117	39.1	173	67.7	104	34.7	779	45.8
Private sector employee	97	19.4	131	43.7	128	42.7	112	37.3	145	48.3	613	36.1
Private business	159	31.8	28	9.3	55	18.3	15	5.0	51	17.0	308	18.1
Father education												
Illiterate	6	1.2	3	1.0	8	2.7	3	1.0	2	0.7	22	1.3
Primary level	52	10.4	24	8.0	10	3.3	9	3.0	12	4.0	107	6.3
Intermediate level	76	15.2	39	13.0	27	9.0	28	9.3	38	12.7	208	12.2
Secondary level	173	34.6	115	38.3	131	43.7	109	36.3	109	36.3	637	37.5
University level	188	37.6	115	38.3	121	40.3	148	49.3	132	44.0	704	41.4
Postgrad level	5	1.0	4	0.67	3	1.0	3	1.0	7	2.3	22	1.28
Father marital status												
Married	496	99.2	295	98.3	298	99.3	300	100.0	295	98.3	1384	81.4
Divorced	4	0.8	5	1.7	2	0.7	0.0	0.0	5	1.7	316	18.6
Child sex												
Male	229	45.8	166	55.3	169	56.3	160	53.3	157	52.3	881	51.8
Female	271	54.2	134	44.7	131	43.7	140	46.7	143	47.7	819	48.2
Child age												
1–2 months	18	3.6	30	10	18	6	14	4.7	18	6	98	5.8
3–4 months	38	7.6	29	9.7	38	12.7	151	50.3	46	15,3	302	17.8
5–6 months	47	9.4	17	5.7	8	2.7	0.0	0.0	38	12.7	110	6.5
7–12 months	158	31.6	87	29	91	30.3	2	0.7	95	31.7	433	25.5
13–18 months	128	25.6	88	29.3	67	22.3	79	26.3	62	20.7	424	24.9
19–24 months	111	22.2	49	16.3	78	26	54	18	41	13.7	333	19.6
Method of delivery												
Vaginal	366	73.2	235	78.3	241	80.3	210	70.0	202	67.3	1254	73.8
Caesarean	134	26.8	65	21.7	59	19.7	90	30.0	98	32.7	446	26.2
Child weight at delivery time												
< 2.5 Kg.	106	21.2	31	10.3	35	11.7	30	10.0	30	10.0	232	13.6
2.5 Kg.	122	24.4	118	39.3	107	35.7	112	37.3	122	40.7	581	34.2
> 2.5 Kg.	272	54.4	151	50.3	158	52.7	158	52.7	148	49.3	887	52.2
Age of the previous child												
1–2 years	27	5.4	38	12.7	19	6.3	43	14.3	28	9.3	155	9.1
3–5 years	195	39.0	130	43.3	122	40.7	164	54.7	148	49.3	759	44.6
> 5 years	118	23.6	49	16.3	56	18.7	56	18.7	41	13.7	320	18.8
No previous child	160	32.0	83	27.7	103	34.3	37	12.3	83	27.7	466	27.4

Almost two third of mothers (73.8%) gave birth naturally (vaginal delivery), while the rest of them (26.2%) gave birth through caesarean delivery. Regarding the surveyed children, the age distributions of female and male children were more or less equal, 881 (51.8%) males and 819 (48.2%) females. Table 1 showed that 1190 (70%) of the children were aged between 7 and 24 months, whereas 510 (30%) of the children were 6 months of age or less. Regarding the age of the previous child, 466 (27.4%) of children were the first child in the family, whereas 914 (53.7%) had previous brothers or sisters aged between one and 5 years. Results of the infant’s birthweight revealed that,1468 (86.4%) of the children their birthweights were 2.5 Kg or more, whereas 232 (13.6%) weighed less than 2.5 Kg.

### Time of initiation and the early initiation of breastfeeding

According to the WHO, early initiation of breastfeeding (EIBF) indicator is defined as the percentage of children born in the last 24 months who were put to the breast within 1 h of birth [4]. Out of 1700 infants studied, 1559 (91.7%) were breastfed and only 8.3% were never breastfed. Breastfeeding initiation was highest (96.7%) among mothers in Dammam, the Eastern region and lowest (83%) was in Hail, the Northern region of KSA. However, Tables 2 and 3 show that 742 (43.6%) of all infants were breastfed within 1 h after birth, i.e. had timely initiation of breastfeeding. Though, 457 (26.9%) of all infants were breastfed between 1 and ≤ 24 h and 360 (21.2%) of them were not breastfed until more than 24 h after birth. The segregation of data by regions revealed that, Southern regions scored the highest timely initiation (62.7%) of breastfeeding among the five regions while the Northern region scored the lowest (26.3%) timely initiation.

**Table 2:** Time of initiation of breastfeeding

	Initiation of breastfeeding EIBF (%)							
	< 1 Hour (less than 1 h.)		1 ≤ 24 h		> 24 Hours		Never breast fed	
Region	*n*	*%*	*n*	*%*	*n*	*%*	*n*	*%*
Northern (Hail): *n = 300*	79	26.3	86	28.7	84	28	51	17
Central (Riyadh): *n = 500*	192	38.4	145	29	115	23	48	9.6
Western (Jeddah): *n = 300*	135	45.0	98	33	52	17	15	5
Eastern (Dammam): *n = 300*	148	49.3	69	23	73	24.3	10	3.3
Southern (Jazan): *n = 300*	188	62.7	59	20	36	12	17	6
Total (KSA): *n = 1700*	742	43.6	457	26.9	360	21.2	141	8.3

**Table 3:** Timely/early initiation and delayed initiation of breastfeeding

	Timely initiation		Delayed initiation		*p* - value
	*n*	%	*n*	%	
Central (Riyadh)	192	38.4	308	61.6	90.44 (< 0.001)
Northern (Hail)	79	26.3	221	73.7	
Eastern (Damam)	148	49.3	152	50.7	
Western (Jeddah)	135	45	165	55	
Southern (Jazan)	188	62.7	112	37.3	
KSA	742	43.6	958	56.4	

The calculated prevalence of the delay in early initiation of breastfeeding showed wide range of variations among the different regions in the Kingdom (Tables 2 and 3). The Western region scored the highest rate (33%) of delay of initiation of breastfeeding for 24 h or less. Other regions showed rates of delay of 28.7% for the Northern; 29% for the Central; 23% for the Eastern; and 20% for the Southern region. As for the extended delay (more than 24 h) was reported in Northern region (28%) followed by the Eastern region (24%), the Central region (23%), the Western region (17%) and the Southern region (12%) with the overall delay rate of 21% calculated for all children in the five regions.

### Reasons for delaying the early initiation of breastfeeding

Table 4 shows the major reasons reported by mothers for delaying the early initiation of breastfeeding for more than 1 h after birth. Results showed that the routine practices in the hospitals of separating the newborn from mother immediately after birth, was the main reason (41%) for the delaying of early initiation of breastfeeding, as stated by the mothers. This was typical in all regions but higher (49 and 44%) in the Northern and Western regions respectively compared to 35–37% in the other regions. Caesarean operation was the second higher (25.9%) reason for the delay of EIBF across the country, however the highest rates were observed in the Western and Southern regions (39 and 27% respectively). Delay because of the caesarean operation ranged between 20 and 24% in the other regions. Other reasons for the delay of early initiation of breastfeeding revealed by the study were mother sickness (10.7%), prelacteal feeding (10.3%), and child sickness (4.6%). Also to a lesser extent, lack of breast milk (2.3%), lack of knowledge about breastfeeding (1.6%) and cultural factors (1.1%).

**Table 4:** Reasons reported by mothers for delaying the initiation of breastfeeding

Reasons	Central (Riyadh): *n = 500*		Eastern (Dammam): *n = 300*		Northern (Hail): *n = 300*		Western (Jeddah): *n = 300*		Southern (Jazan): *n = 300*		KSA: *n = 1700*	
	*n*	%	*n*	%	*n*	%	*n*	%	*n*	%	*n*	%
Delay at the place of delivery	114	37	72	36	130	49	82	44	44	35	442	40.9
Caesarean delivery	74	24	39	20	60	23	73	39	34	27	280	25.9
Mother sickness	38	12	23	12	31	12	10	5	14	11	116	10.7
Child given prelacteal feed	4	1	39	20	30	11	15	8	23	18	111	10.3
Cultural factors	6	2	4	2	2	1	0	0	0	0	12	1.1
Child sickness	29	9	10	5	4	2	3	2	4	3	50	4.6
No reasons	28	9	0	0	0	0	0	0	0	0	28	2.6
Lack of breast milk	8	3	8	4	5	2	2	1	2	2	25	2.3
Lack of knowledge of breastfeeding	7	2	3	2	1	0.4	1	0.5	5	4	17	1.6
Total	308	100	198	100	263	100	186	100	126	100	1081	100

Cultural practices that involve discarding colostrum, feeding newborns supplemental foods or drinks, having a health professional routinely give the newborn a specific liquid such as sugar water or infant formula, were all investigated among the mothers. Results (Table 5) indicated that only 47% of the newborn babies have been fed colostrum. Colostrum feeding was highest (65%) among newborns of the Southern region and lowest (25%) in the Northern region. More importantly, 51% of the newborn babies were found to be fed infant formula as the first feed. This practice was mainly performed by health personnel with the hospital delivery.

**Table 5:** Newborn first feed

Region	Newborn first feed						Chi-square (*p* - value)
	Breast milk		Infant formula		Glucose water		
	*n*	%	*n*	%	*n*	%	
Central (Riyadh)	174	38.4	320	64	6	1.2	167 (< 0.001)
Northern (Hail)	75	25	215	71.3	10	33	
Eastern (Dammam)	177	59	120	40	3	1	
Western (Jeddah)	172	57.3	120	40	8	2.3	
Southern (Jazan)	196	65.3	97	32.3	7	2.3	
KSA	794	46.7	872	51.3	34	2	

### Determinants of the early initiation of breastfeeding in KSA

A significant difference was observed in early initiation of breastfeeding between mothers who delivered vaginally 633 (85%) and those delivered by caesarean section 109 (15%), between mothers who knew about the right time for early initiation of breastfeeding 630 (85%) and those who didn’t know 112 (15%), between mothers who received information about breastfeeding 524 (71%) and those who didn’t receive any breastfeeding information 218 (29%), and finally, between mothers who were resident of the different regions of the kingdom (38, 26, 61, 45 and 63%) for the Central, Northern, Eastern, Western and the Southern regions respectively (Table 6).

**Table 6:** Determinants of timely initiation of breastfeeding: degree of association

Parameter	Early initiation of breastfeeding (*n* = 1700)		*p* – value (Chi-square)
	Delayed initiation	Timely initiated	
Location			
Urban	776 (45.7%)	584 (34.4%)	1.378 (0.241)
Rural	182 (10.7%)	158 (9.3%)	
Method of delivery			
vaginal	621 (36.5%)	633 (37.2%)	90.6 (0.001)
Caesarean	337 (19.8%%)	109 (6.4%)	
Received antenatal care			
Yes	886 (52.1%)	693 (40.8%)	0.526 (0.468)
No	72 (4.2%)	49 (2.9%)	
Received postnatal care			
Yes	823 (48.4%)	636 (37.4%)	0.013 (0.91)
No	135 (7.9%)	106 (6.2%)	
Previous child (child order)			
No previous child	286 (16.8%)	180 (10.6%)	6.58 (0.01)
Second child and above	672 (39.5%)	562 (33.1%)	
Knowledge about timing of breastfeeding initiation			
Don’t know when to initiate breastfeeding	884 (52%)	112 (6.6%)	1026.6 (< 0.001)
Know when to initiate breastfeeding	74 (4.4%)	630 (37.1%)	
Infant’s mother educational level			
Secondary school level and below	456 (26.8%)	400 (23.5%)	6.65 (0.01)
Graduate and postgraduate levels	502 (29.5%)	342 (20.1%)	
Child mother age			
< 25 year	146 (8.6%)	124 (%)7.3	0.678 (0.41)
≥ 25 years	812 (47.8%)	618 (36.4%)	
Child weight at delivery time			
< 2.5 Kg	161 (9.5%)	71 (4.2%)	18.58 (< 0.001)
≥ 2.5 Kg	797 (46.9%)	671 (39.5%)	
Receiving breastfeeding information			
Yes	551 (32.4%)	524 (30.8%)	30.8 (< 0.001)
No	407 (23.9%)	218 (12.8%)	
Child mother occupation			
Working woman	340 (20%)	230 (13.5%)	3.79 (0.052)
Housewife	618 (36.4%)	512 (30.1%)	
Region			
Central (Riyadh)	308 (18.1%)	192 (11.3%)	90.45 (< 0.001)
Northern (Hail)	221 (13%)	79 (4.7%)	
Eastern (Dammam)	152 (8.9%)	148 (8.7%)	
Western (Jeddah)	165 (9.7%)	135 (7.9%)	
Southern (Jazan)	112 (6.6%)	188 (11.1%)	

On the other hand, five variables showed no significant difference (Table 6) in practicing initiation of breastfeeding (timely vs delayed). These variables were: infant birthweight (< 2.5 kg vs ≥ 2.5 kg), antenatal and postnatal care, mother age (< 25 years vs ≥ 25 years), mother work (working mothers and student vs housewives) and residence location (urban vs rural).

Additionally, multivariable logistic regression, was performed to assess determinants related to early initiation of breastfeeding (Table 7). It is clearly evident that, early initiation of breastfeeding was significantly associated with the mode of delivery (vaginal vs caesarean) (AOR 2.071; 95% CL 1.43, 2.99) i.e. mothers who had a caesarean delivery had about 2.07 times higher odds for delaying breastfeeding by > 1 h when compared to mothers who had a vaginal delivery. Moreover, significant association was also observed between early initiation of breastfeeding and mother educational level (AOR 1.54; 95% CL 1.13, 2.11); uninformed mothers regarding the right time for the early initiation of breastfeeding (AOR 0.012; 95% CI 0.0028, 0.017) and mothers who received breastfeeding information during pregnancy (AOR 1.49; 95% CI 1.026, 2.54).

**Table 7:** Multivariable logistic regression parameter estimates of early initiation of breastfeeding

Parameter	Β	Adjusted Odds Ratio (95% Confidence Interval)
Method of delivery		
Vaginal	0. 728^a^ (0.188)	2.071 (1.43, 2.99)
Caesarean	0	1
Knowledge about timing of breastfeeding initiation		
Don’t know when to initiate breastfeeding	−4.442^a^ (0.191)	0.012 (0.008, 0.017)
Know when to initiate breastfeeding	0	1
Maternal educational level		
Secondary school level and below	0.432^a^ (0.161)	1.541 (1.13, 2.11)
Graduate and postgraduate levels	0	1
Breastfeeding information		
Yes	0.397^a^ (0.189)	1.487 (1.026, 2.54)
No	0	1
Region		
Middle	0.944^a^ (0.259)	2.57 (1.55, 4.27)
Northern	0.621^a^ (0.292)	1.86 (1.05, 3.297)
Eastern	0.705^a^ (0.276	2.03 (1.18, 3.45)
Western	0.891^a^ (0.78)	2.437 (1.41, 4.12)
Southern	0	1

^a^significant at 5% level of significance or less

## Discussion

General characteristics of the study participants such as age distributions of female and male children (52/48%), multiparity (73%), vaginal as a common mode of delivery (73.8%), and a normal birthweight (86.4%) were similar to the results of the Saudi population (2016) reported by the General Authority of Statistics of KSA [2].

In this study only 8.3% of mothers did not initiate breastfeeding, this is nearly similar to 8.1% rate reported by El Gilany [27], however earlier studies in KSA reported rates of never breastfeeding ranged from 1.4 to 13.1% [21, 25, 30–33]. The high prevalence of breastfeeding initiation observed in the current study (91.7%) denotes the inclination of Saudi mothers to breastfed, a fact that was also reached by other investigators [3, 26, 27, 34, 35]. Previous studies showed similar initiation rates of 95% in Riyadh [23], 80.2% in Mecca [28], 90% in Western region [35], 91.9% in Al-Hassa (Eastern region) [27] and 87.7% in Central region [23, 36]. The prevalence of breastfeeding initiation showed variation between the different region of the country where, Eastern, Western and Southern regions reported the highest initiation rates of 96.7, 95 and 94% respectively, and 90% for the Central region, while the Northern region reported the lowest (83%) initiation rates. Similar results were obtained by Al-Hreashy et al. [36].

Despite the high breastfeeding initiation rate of 91.7%, the early initiation of breastfeeding (within 1 h after birth) for the country (44%), considered as a “fair” score according to the WHO criteria. The sub-regional data showed variations from a “good score” of 63% in the Southern region, followed by “fair scores” of 49.3, 45 and 38.4% in each of the Eastern, Western and Central regions respectively. While, the lowest “poor score” of 26.3% was reported in the Northern region. This rate of 44% EIBS is higher than the 11.4 and 23.2% obtained by El-Gilany et al. [27] and Al-Mouzan et al. [25] respectively, though, it is similar to the EIBF rates reported by UNICEF [37] in Pakistan, India, Bangladesh and Nepal (29, 41, 47 and 45% respectively). Moreover, this result of 44% is not far from the 38.1% result obtained by Azzeh et al. in Mecca region [28] and the 51.3% reported by Alwelaie in Riyadh [23]. In contrast a low EIBF rate of 16.7% was reported by Albokhary in Jeddah, [38], 22% reported in Taif [39, 40] and 21.6% reported by Al-Hreashy in Dammam [36].

The overall delay in EIBF of 27 and 21% for less than 24 h. and that beyond 24 h. respectively, were similar to the results (28.1%) obtained by El Mouzan et al. [25] for delayed initiation of beyond the first 6 hrs. of birth. However, Azzeh et al. [28], reported 42.7% delay in the initiation of breastfeeding beyond 1 h after birth in Western region of KSA.

Factors associated with the compliance with the EIBF (≤ 1 h. after birth) found in the present study were the mode of delivery (50% for natural vs 24% caesarean delivery), residential location (46% for rural vs 43% urban residence), child birthweight (31% for low birthweight children, 49% for normal birthweight children and 43% for children with birthweight ≥2.5 Kg.), and child order (39% one child vs 46% the second child and more).

The significant effect of caesarean section was indicated by the high delay rate in EIBF among KSA mothers in the present study. Results of the effect of caesarean section were consistent with those obtained by Azzeh et al. [28], in Mecca, and that obtained by Alzaheb in Tabuk region in KSA [8] and with other previous studies in KSA [38, 40], which recognized caesarean section as a risk factor for delaying breastfeeding initiation.

In consistent with results obtained by Gilany [27], this study revealed that mothers with higher parity (46%) were more likely to initiate breastfeeding within the first hour of birth. However, and contradicting to Badaya et al. and Mathur et.al [41, 42], results of this study revealed that, rates of timely initiation of breastfeeding is higher (49%) among children with low birthweight (< 2.5 Kg.),

The main reported reason for delaying early initiation of breastfeeding beyond 1 h of birth was the delay from the place of delivery (40.9%) i.e. mothers and babies were separated immediately after birth and the newborn brought back to mother after more than 1 h after delivery. Similar findings were reported in KSA by Ogbeide et al. [6], Mosher [43], Azzeh et al. [28], Fida [35] and in Greece by Daglas et al. [42] and Mathur et al. [44] in India.

This delay highlights practices at the place of delivery, and raises the issue of suitable hospital policies and staff training be implemented to support mothers in timely initiating breastfeeding, and to discourage free distribution of breast milk substitutes in hospital, all have a significant relationship with early initiation of breastfeeding [8, 23, 45, 46]. In this study, giving prelacteal feed was reported by 10.3% of mothers as a reason for delaying the early initiation of breastfeeding. This is similar previous studies [8, 27, 47, 48] who also suggested that prelacteal feeding may result in lactation failure [27] and weakens the infant suckling stimulus [48].

The high consumption of infant formula among > 50% of mothers reported in this study could be related to breastfeeding practices in KSA such as, the low rate (less than 10%) of exclusive breastfeeding up to 6 months of age [19, 24–26], and the partial breastfeeding that became a trend for feeding in the country [25, 26, 36]. In addition to the non-compliance with the International Code of Marketing Breast Milk Substitutes [19, 25, 28], and the lack of strict monitoring system for registration and marketing of infant formulas from the Saudi authorities [39, 43].

### Determinants of the early initiation of breastfeeding in KSA

The results of the chi-square test (*p* - value of < 0.05) and the multivariant analysis indicated that women who gave birth vaginally were more likely to timely initiate breastfeeding than those who had a caesarean section (AOR 2.071; 95% CL 1.43, 2.99). These results are consistent with previous studies in KSA [34, 35, 39, 40], which identified caesarean section as a risk factor for breastfeeding initiation. This study has identified another four factors as having statistically significant associations with early initiation of breastfeeding. The first positively associated factor is the knowledge of the mother about the right time for early initiation (AOR 0.012; 95% CI 0.0028, 0.017). The other significantly associated factors with the early initiation, were receipt of breastfeeding information during pregnancy (AOR 1.49; 95% CI 1.026, 2.54), educational level of the mother (AOR 1.54; 95% CL 1.13, 2.11) and finally, the region of residence (Central, Northern, Southern, Eastern and Western).

## Conclusions

The main determinant of the early initiation of breastfeeding were the infants’ mothers: who give birth by caesarean section, educational level (graduates and above); who didn’t receive breastfeeding information; unknowledgeable about the right time of breast feeding as well as the residence region.

The key finding of this study is that the initiation of breastfeeding is high while, the early or timely breastfeeding initiation (within 1 h of birth) is considered fair according to the World Health Organization classification. However, the willingness of Saudi mothers to breastfeed indicated by the high prevalence of breastfeeding initiation, must be supported by effective government policy regarding breastfeeding support and marketing of breast milk substitutes. Moreover, behavior change strategies, are needed to encourage mothers and families to demand support for the early initiation of breastfeeding from health personnel. Nonetheless, links between health facilities and communities need to be strengthened, and community networks that protect, promote and support breastfeeding should be encouraged.

## Abbreviations

AOR: Adjusted Odd Ratio
EIBF: Early initiation of breastfeeding
EMRO: Eastern Mediterranean Regional Office
FMOH: Federal Ministry of health
IRB: Institutional review board
IYCF: Infant and young child feeding
KACST: King Abdul-Aziz city for science and technology
KSA: Kingdom of Saudi Arabia
KSU: King Saud University
MENA: Middle East and North Africa
MOH: Ministry of health
NSTIP: National science, technology and innovation plan
PHCCs: Primary health care centers
UNICEF: United Nations Children’s Fund
WBTi: World Breastfeeding Trend initiative
WHO: World Health Organization
